# Evidence for dynamic resource partitioning between two sympatric reef shark species within the British Indian Ocean Territory

**DOI:** 10.1111/jfb.13938

**Published:** 2019-04-11

**Authors:** David J. Curnick, Aaron B. Carlisle, Matthew J. Gollock, Robert J. Schallert, Nigel E. Hussey

**Affiliations:** ^1^ Institute of Zoology Zoological Society of London, Regents Park London UK; ^2^ Centre for Biodiversity and Environment Research, Department of Genetics, Evolution and Environment University College London London UK; ^3^ School of Marine Science and Policy University of Delaware Lewes Delaware USA; ^4^ Hopkins Marine Station Stanford University Pacific Grove California USA; ^5^ Biological Sciences University of Windsor Windsor Ontario Canada

**Keywords:** British Indian Ocean Territory, reef shark, resource partitioning, stable isotope, δ^13^C, δ^15^N, δ^34^S

## Abstract

Stable‐isotope analyses (δ^13^C, δ^15^N and δ^34^S) of multiple tissues (fin, muscle, red blood cells and plasma), revealed ontogenetic shifts in resource use by grey reef sharks *Carcharhinus amblyrhynchos* and resource partitioning with silvertip sharks *Carcharhinus albimarginatus* within the British Indian Ocean Territory marine protected area (MPA). Resource partitioning varied temporally, with *C. albimarginatus* feeding on more pelagic prey during October to January, potentially attributable to an influx of pelagic prey from outside the MPA at that time. Reef sharks may therefore be affected by processes outside an MPA, even if the sharks do not leave the MPA.

1

Sharks are believed to play an important role in structuring marine communities and consequently contribute to ecosystem productivity (Heithaus *et al.,* 2008). However, the ecological role of reef sharks has been at the forefront of recent debate (Heupel *et al.,* 2014; Rizzari *et al.,* 2014; Roff *et al.,* 2016) as they often share similar habitats and trophic roles (Speed *et al.,* 2012), but have been shown to exhibit resource partitioning (Rizzari *et al.,* 2014). Some authors have classified reef sharks as top predators (Friedlander & DeMartini, 2002), whereas others suggest they are mesopredators (Heupel *et al.,* 2014) with a high degree of functional redundancy within the guild (Frisch *et al.,* 2016b). To understand these relationships and their ecological importance better, interactions such as resource partitioning need to be examined.

Stable‐isotope analysis (SIA) of nitrogen (δ^15^N) and carbon (δ^13^C) provide insights into trophic interactions and resource partitioning (Papastamatiou *et al.,* 2006; Plumlee & Wells, 2016), which are key for understanding how ecological communities are structured. Previous studies of sharks have shown that the turnover rates of stable isotopes in muscle and fin tissue are relatively slow, incorporating dietary information over more than a year (MacNeil *et al.,* 2006). In contrast, red blood cells (RBC) turnover faster (minimum 95% turnover rate estimates of 258 days for δ^15^N and 405 days for δ^13^C) and plasma quicker still (170 days for δ^15^N and 252 days for δ^13^C for plasma; Caut *et al.,* 2013) and thus represent diets over shorter time scales. Thus, SIA can reveal temporal changes in the trophic ecology of organisms if multiple tissue types are sampled (Hussey *et al.,* 2012).

In this study, SIA was used to examine resource partitioning and seasonal variation in resource use, specifically if dynamic resource partitioning occurs between two sympatric reef sharks, the grey reef shark *Carcharhinus amblyrhynchos* (Bleeker 1856) and the silvertip shark *Carcharhinus albimarginatus* (Rüppell 1837), within the British Indian Ocean Territory (BIOT) marine protected area (MPA). To complement carbon and nitrogen SIA, sulphur (δ^34^S) was also examined. Few δ^34^S data exist in truly marine environments but, as seawater (δ^34^S *c*. 22.9‰) and sediments (δ^34^S *c*. 1‰) have distinct δ^34^S signatures within the marine environment, it can help discriminate between organisms feeding on pelagic (expected higher δ^34^S values) *v*. benthic and lagoon‐dependent prey (expected lower δ^34^S values; Gajdzik *et al.,* 2016). Although previously used to investigate the feeding ecology of coastal sharks (Plumlee and Wells 2016), these data represent the first application of δ^34^S data to understand reef shark ecology.

All procedures undertaken in the course of this study were approved by the Zoological Society of London ethics committee.

Sampling was conducted between the 17th and 23rd January 2015 around Diego Garcia within the BIOT. Teleost fishes were caught using baited hand‐lines, measured (fork length, *L*_F_) and muscle tissue extracted from below the dorsal fin. Sharks were caught using baited hand‐lines or short long‐lines (<20 hooks) with barbless circle hooks that were checked hourly. Once caught, species, sex and morphological measurements (*L*_F_ and pre‐caudal length, *L*_PC_) were recorded and samples of first dorsal fin, muscle (at the base of the first dorsal fin), and blood were collected before returning the sharks to the water. Blood samples were taken from the caudal vein using a heparinized needle and syringe. Blood samples were spun immediately in a portable centrifuge until the blood fractionated. The resultant plasma and RBC layers were separated by pipette. Samples were stored on ice during transport, immediately frozen (−20°C) on return to the laboratory, and then processed and analysed for δ^13^C, δ^15^N and δ^34^S following standard procedures (Supporting Information Material and Methods and Table S1).

The relationship between δ^13^C, δ^15^N and δ^34^S and _LPC_ were analysed using least‐squares linear regressions. Differences in isotope values between species were compared using ANOVA (α = *P* < 0.05). Maximum likelihood standard ellipses were created for the δ^13^C and δ^15^N values for all tissues and for both shark species following Jackson *et al*. (2011). Ellipses were also generated for δ^34^S against both δ^13^C and δ^15^N for shark and teleost muscle tissue. In addition to standard ellipse area (SEA; contain *c*. 40% of the data and represent the core isotopic niche) and standard ellipse areas corrected for small sample size (SEAc), traditional convex hulls were also estimated (Layman *et al.,* 2007). The overlap between *C. amblyrhynchos* and *C. albimarginatus* SEAc ellipses was calculated as a proportion of total SEAc ellipse area for each tissue type. Ellipse sizes were compared between species using Bayesian inference techniques within the *jags* and *SIAR* packages in R 3.4.0 (http://www.r-project.org). Bayesian carbon isotope mixing models were used to quantify the probable contribution of reef *v*. pelagic derived food items to the diet of both shark species. End members were set as the most ^13^C depleted (pelagic) and most ^13^C enriched (reef) of the teleosts sampled (Table 1). The statistical programme R 3.4.0 was used for all analyses.

**Table 1:** Stable–isotope (δ^13^C, δ^15^N and δ^34^S) values of different tissue types and morphometric measurements of *Carcharhinus amblyrhynchos*, *Carcharhinus albimarginatus* and Potential competitor–prey teleost species. Bold denotes significant difference between species: P < 0.05 (see Supporting Information Table S2 for diagnostics)

Species	*L*_PC_ (cm)	*L*_F_ (cm)	n	Fin		Muscle			RBC^a^		Plasma^b^	
	Mean	Mean	(M:F)	δ^13^C‰	δ^15^N‰	δ^13^C‰	δ^15^N‰	δ^34^S‰	δ^13^C‰	δ^15^N‰	δ^13^C‰	δ^15^N‰
	(range)	(range)		(mean ± SD)	(mean ± SD)	(mean ± SD)	(mean ± SD)	(mean ± SD)	(mean ± SD)	(mean ± SD)	(mean ± SD)	(mean ± SD)
Sharks												
*C. amblyrhynchos*	102.4 (55–125)	113.5 (58–138)	15 (0:15)	**−12.2 ± 1.1**	12.3 ± 0.4	**−13.4 ± 1.7**	**14.0 ± 0.6**	17.5 ± 0.9	**−14.0 ± 1.3**	**13.7 ± 0.4**	**−13.7 ± 1.0**	**13.1 ± 0.3**
*C. albimarginatus*	111.4 (91–141)^c^	124.5 (103–156)^c^	11 (7:4)	**−13.7 ± 0.3**	12.0 ± 0.4	**−14.6 ± 1.0**	**13.3 ± 0.8**	17.0 ± 2.4	**−16.2 ± 0.2**	**13.2 ± 0.2**	**−15.6 ± 0.2**	**12.4 ± 0. 2**
Teleosts												
Barracuda, Sphyraenidae		57.7 (45.5–72)	10			−17.0 ± 0.5	13.6 ± 0.5	15.0 ± 1.7				
Grouper, Serranidae		61.5 (21–95)	4			−15.3 ± 1.8	13.7 ± 0.4	13.9 ± 5.4				
Snapper, Lutjanidae		36.3 (21–59)	3			−16.8 ± 0.2	13.1 ± 0.2	16.2 ± 1.8^d^				
Trevally, Carangidae		54.0 (51–57)	2			−13.6 ± 2.0	13.7 ± 0.1	17.2 ± 0.8				
Tuna, Scombridae		52.6 (30–70)	12			−17.1 ± 0.5	13.1 ± 1.1	16.6 ± 2.8				
Wahoo, Scombridae		110.5 (94–133)	6			−16.7 ± 0.2	12.5 ± 0.7	17.1 ± 1.0				

*L*_PC_, pre–caudal length; *L*_F_, fork length; *n*, the numbers of animals sampled with shark sex ratios (M:F); RBC, red blood cells.

a RBC samples were 5 *C. amblyrhynchos* and 7 *C. albimarginatus* only.

b Plasma samples were collected for 5 *C. amblyrhynchos* and 7 *C. albimarginatus* only.

c Morphometric measurements of one male *C. albimarginatus* were not recorded.

d Sulphur data were not available for one tuna.

Twenty‐six sharks were sampled, 15 *C. amblyrhynchos* and 11 *C. albimarginatus*, along with 37 Potential competitor–prey species spanning five teleost families (barracuda, Sphyraenidae; grouper, Serranidae; snapper, Lutjanidae; trevally, Carangidae; tuna, Scombridae; wahoo, Scombridae; Table 1). Mean (± SD) *C. amblyrhynchos L*_PC_ = 102.4 ± 22.23 cm; all individuals were female. *C. albimarginatus L*_PC_ = 111.4 ± 18.18 cm and included seven males and four females (Table 1). *L*_PC_ correlated positively with δ^13^C in *C. amblyrhynchos* fin (*P* < 0.05, *r* ^2^ = 0.44) and muscle tissue (*P* < 0.05, *r* ^2^ = 0.55) and δ^15^N of fin tissue (*P* < 0.05, *r* ^2^ = 0.61), indicating an ontogenetic shift in foraging location and trophic position, respectively (Table 2 and Supporting Information Figures S1, S2). In contrast, no significant relationships were found between length and δ^13^C or δ^15^N in *C. albimarginatus* for any tissue type suggesting that neither foraging location nor trophic position were influenced by body size (Table 2 and Supporting Information Figure S2). Endo *et al*. (2016) reported similar results from *C. albimarginatus* in the Pacific Ocean, although they noted a decline in δ^13^C with increasing body size in females. No significant length relationships were found for either species in relation to δ^34^S (Table 2 and Supporting Information Figure S3), though the small sample size may affect observed relationships.

**Table 2:** Results of linear regressions examining the effect of size of reef sharks on δ^13^C, δ^15^N and δ^34^S values in fin, muscle, red blood cells (RBC) and plasma in reef sharks. *C. amblyrhynchos* with pre‐caudal lengths (PCLs) less than 80 cm in length were removed due to potential maternal effects

Species	δ^13^C			δ^15^N			δ^34^S		
	r^2^	*P*	slope	r^2^	*P*	slope	r^2^	*P*	slope
*C. amblyrhynchos*									
Fin	**0.48**	**0.01**	**0.06**	**0.64**	**<0.001**	**0.03**			
Muscle	**0.58**	**<0.001**	**0.09**	0.01	0.81	0.00	0.16	0.16	−0.02
RBC	0.85	0.08	0.19	0.46	0.32	0.03			
Plasma	0.76	0.13	0.13	0.32	0.44	0.02			
*C. albimarginatus*									
Fin	0.08	0.36	0.00	0.17	0.16	−0.01			
Muscle	0.06	0.50	0.01	0.02	0.66	−0.01	0.10	0.39	0.04
RBC	0.01	0.87	0.00	0.46	0.14	0.01			
Plasma	0.34	0.22	−0.01	0.10	0.55	0.00			

Bold denotes significant relationships with PCL at *P* ≤ 0.05.

*C. amblyrhynchos* had significantly higher δ^15^N values in all tissues except fin when compared with *C. albimarginatus*, but differences were relatively small (0.5–0.7‰; Table 1 and Supporting Information Table S2). This supports previous studies that have found these two species feed at a similar trophic level (Cortés, 1999). *C. amblyrhynchos* also had significantly higher δ^13^C values than *C. albimarginatus* in all tissues (+1.2–2.2‰; Table 1 and Supporting Information Table S2). This indicates that while these species co‐exist and feed at a similar trophic level, there is a degree of resource partitioning. Overall, δ^15^N values of teleosts sampled were similar to those of both *C. amblyrhynchos* and *C. albimarginatus*, suggesting these species may not represent direct prey for reef sharks and feed at similar trophic levels. These data broadly support observations of Roff *et al*. (2016) who classify *C. amblyrhynchos* as mesopredators that occupy a similar trophic level to large piscivorous fishes. Owing to restrictions in the BIOT MPA, it was not possible to sample lower trophic level species that have been shown to contribute significantly to the diet of reef sharks (Frisch *et al.,* 2016b). Tunas have the lowest mean δ^13^C values and therefore the strongest pelagic signal (mean ± SD = −17.1% ± 0.5); whereas the highest mean δ^13^C values were found in reef associated trevally (−13.6‰ ± 2.0; Table 1).

We used the δ^13^C of potential competitor–prey teleost species to assess the relative importance of different food webs (pelagic *v*. reef) to sharks. To do this we used a δ^13^C discrimination factor of 0.00‰ and a standard deviation of ± 0.33 (Hussey *et al.,* 2010). Bayesian mixing models indicate that *C. amblyrhynchos* derive *c*. 78% of their biomass from reef sources compared with just *c*. 60% for *C. albimarginatus* (Figure 1). Interestingly, while the relatively increased dependence of *C. amblyrhynchos* on reef resources presented here are broadly consistent with findings for *C. amblyrhynchos* in the Great Barrier Reef (Frisch *et al.,* 2016b), they are in contrast to *C. amblyrhynchos* in Palmyra Atoll (5° 53′ 01″ N, 162° 04′ 42″ W) which derive the majority (*c*. 86%) of their biomass from pelagic sources (McCauley *et al.,* 2012). These data suggest significant ecological variability within a reef‐shark species dependent on location.

**Figure 1:**
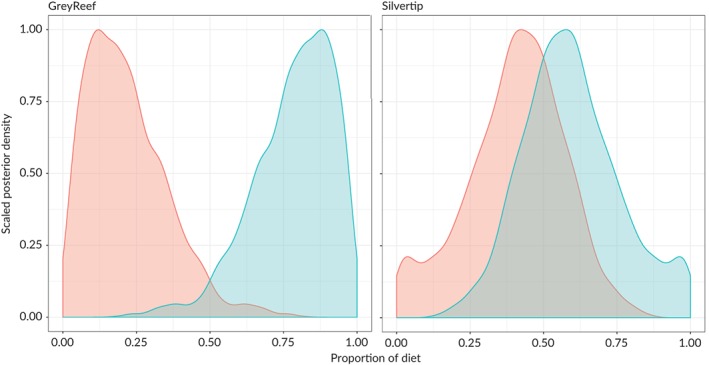
Bayesian isotope mixing models were used to determine the extent that *Carcharhinus amblyrhynchos* and *Carcharhinus albimarginatus* were reliant on reef (blue) or pelagic (red) resources. End members were set as the most δ^13^C depleted (pelagic) and most δ^13^C enriched (reef) of the teleosts sampled (trevally (Carangidae) for reef, tuna (Scombridae) for pelagic). Posterior probability distributions indicate model predictions of reliance on a given source with higher values indicating greater reliance

Here, some of the first δ^34^S data examining trophic interactions and resource partitioning among sharks on remote coral reefs is presented. No significant difference in δ^34^S values was observed between shark species, but both exhibited large ranges (*C. amblyrhynchos* 15.4–18.6‰; *C. albimarginatus* 11.8–19.0‰; Table 1 and Figure 2e,f). Mean δ^34^S values for reef sharks (17.0–17.5‰) suggest they are heavily dependent on pelagic productivity (Gajdzik *et al.,* 2016). However, the wide ranges suggest they have diverse intraspecific feeding strategies, especially *C. albimarginatus*, verifying previous dietary studies (Cortés 1999). Teleosts had similar δ^34^S values when compared with reef sharks, again suggesting a strong dependence on pelagic prey items (Table 1). Interestingly, epinephelids had the lowest mean δ^34^S value (13.9 ± 5.4‰) and the largest range (Table 1). This is consistent with their broad diet across benthic and pelagic sources (Frisch *et al.,* 2016a).

**Figure 2:**
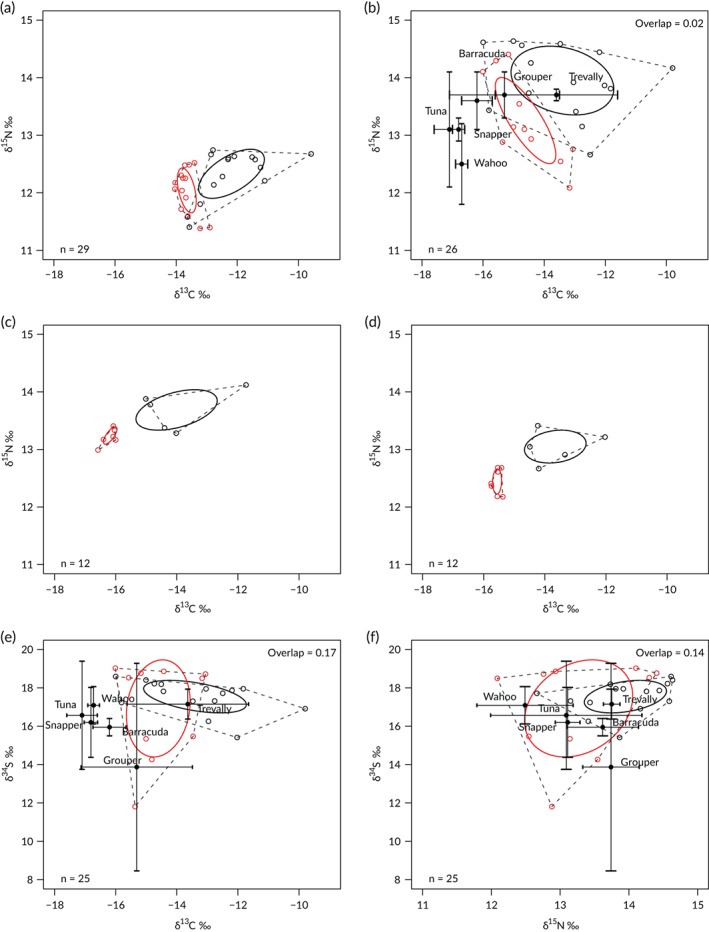
(a) Maximum likelihood standard ellipse areas (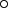 , 40% of the data) for isotopes δ^13^C *v*. δ^15^N in fin, (b) muscle, (c) red blood cell, (d) plasma and for isotope δ^34^S *v*. δ^15^C (e) and δ^15^N (f) of *Carcharhinus amblyrhynchos* (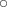) and *Carcharhinus albimarginatus* (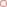). Convex hulls (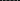) are drawn between the centers of each group. Overlapping values, if present, are the proportion of overlapping area of the two ellipses. Potential competitor–prey teleost data are shown (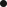) with associated error bars (± 1 SD). Ellipses for red blood cell and plasma presented for reference but represent small sample sizes (< 10) and therefore come with lower confidence

For all tissue types, SEAc for δ^13^C and δ^15^N values were considerably larger in *C. amblyrhynchos* (fin = 1.26; muscle = 3.30; RBC = 1.75; plasma = 1.18) than *C. albimarginatus* (fin = 0.36; muscle = 1.57; RBC = 0.08; plasma = 0.12; Figure 2a–d and Supporting Information Table S3), suggesting *C. amblyrhynchos* have a larger isotopic niche and a more generalised feeding ecology than *C. albimarginatus*. Overall, very little isotopic niche overlap occurred between the two species with only muscle tissue showing a small degree of overlap (0.02; Figure 2b, but note small sample sizes). These data indicate clear niche partitioning between these two closely related species. Our findings also support the categorization proposed by Frisch *et al*. (2016b) that *C. amblyrhynchos* are true reef sharks but *C. albimarginatus* are less dependent on reef‐based resources. Furthermore, it appears that this partitioning of resources varies temporally. While muscle isotope data indicate that these sharks exploit different resources over long time frames, niche partitioning was most evident in tissues with faster turnover rates (Figure 2a–d). Though our sample size was limited, we postulate that this may be driven by seasonal increases in the use of pelagic resources by *C. albimarginatus* relative to *C. amblyrhynchos*. As sampling occurred in January, this would suggest that *C. albimarginatus* increased their use of pelagic resources in prior months (*c*. October–December). This timeframe matches the historical peak pelagic fishing season (October–January) within BIOT when a purse‐seine fishery targeted migrating yellowfin tuna *Thunnus albacares* (Bonnaterre 1788) (Mees *et al.,* 2009). Recent tagging studies have shown that *C. albimarginatus* remain within the BIOT MPA boundaries (A Carlisle unpubl. data) and are therefore directly spatially protected from the effects of legal fishing vessels, although the illegal poaching of sharks occurs within the MPA (Ferretti *et al.,* 2018). Such findings are important to consider when establishing and managing protected areas, which are usually focused on protecting intrinsic characteristics of a species such as home ranges or important life cycle events (*e.g*., spawning grounds; Green *et al.,* 2014). However, it is clear that relative to *C. amblyrhynchos*, *C. albimarginatus* are more dependent on pelagic prey, some of which may migrate into BIOT MPA from outside. Thus, sharks, particularly *C. albimarginatus*, within the MPA may therefore be indirectly affected by processes such as fisheries beyond its borders.

## Supporting information


**File S1.**
