# Supplementary material for: Evidence for dynamic resource partitioning between two sympatric reef shark species within the British Indian Ocean Territory
Source: J Fish Biol. 2019 Apr 11;94(4):680–5. doi: 10.1111/jfb.13938 (PMC6849741; doi:10.1111/jfb.13938)
Supplement: Supplementary file 1 — File S1. [file JFB-94-680-s001.docx]

**Supporting Information**

**Supplementary methodological and analytical information for Curnick et al. 2019**

**MATERIAL AND METHODS**

**Sample preparation and stable isotope analysis (SIA - δ^13^C, δ^15^N and δ^34^S)**

All shark and teleost samples were lyophilised, homogenised to a fine powder and then lipid extracted following standard chloroform-methanol procedures (Hussey *et al*., 2012). For shark muscle tissue, urea extraction was undertaken using water rinsing following Li *et al*., (2016) and Carlisle *et al*., (2016). Approximately 400–600 μg of tissue was then weighed into tin cups. For sulphur, all samples and standards were thoroughly mixed with 2000–3000 μg of vanadium pentoxide within the tin capsules to promote sulphur combustion.

Following preparation, all samples were analysed for carbon, nitrogen and sulphur using an elemental analyser (Costech 4010; www.costechanalytical.com) interfaced to a Thermo Finnigan Delta^PLUS^ mass spectrometer (www.thermofisher.com).

Stableisotope values are expressed in delta (δ) values as the ratio of an unknown sample to a recognized standard in parts per thousand (per mil, ‰), δ^13^C, δ^15^N or δ^34^S = 1000 [(*R*_sample_ / *R*_standard_) – 1], where *R*_sample_ and *R*_standard_ are the isotope ratio (^13^C/^12^C, ^15^N/^14^N or ^34^S/^32^S) of the sample and standard, respectively.

**Accuracy and precision of carbon (δ^13^C), nitrogen (δ^15^N) and sulphur (δ^34^S) stable-isotope analyses**

Precision was assessed by the SD of replicate analyses of four standards; NIST1577c, internal lab standard (tilapia muscle), USGS 40 and Urea (*n* = 15 for all), measured ≤ 0.16‰ for δ^15^N and ≤ 0.15‰ for δ^13^C for all standards. For δ^34^S, precision was ≤ 0.28‰ based on replicate analyses of four standards; internal lab standard (tilapia muscle), NIST 1577c, NIST 8554 and NIST 8555.

  Accuracy was based on the certified values of USGS 40 (*n* = 15 for δ^13^C) and Urea IVA33802174 (*n* = 15 for δ^15^N) analysed throughout runs and not used to normalize samples showed a difference of -0.13‰ for δ^15^N and -0.08‰ for δ^13^C from the certified value. For δ^34^S, a difference of 0.28‰ was measured versus certified values of USGS 42 (n=15).

**REFERENCES**

Hussey NE, Olin JA, Kinney MJ, McMeans B, Fisk AT (2012) Lipid extraction effects on stable isotopes (δ^15^N and δ^13^C) in elasmobranch muscle tissue. *Journal of Experimental Marine Biology and Ecology* 434-435, 7-15.

Li Y, Zhang Y, Hussey NE, Dai X (2016) Urea and lipid extraction treatment effects on δ^15^N and δ^13^C values in pelagic sharks. *Rapid Communications in Mass Spectrometry*, 29, 1-8.

Carlisle AB, Litvin SY, Madigan DJ, Lyons K, Bigman JS, Ibarra M, Bizzarro JJ (2016) Interactive effects of urea and lipid content confound stable isotope analysis in elasmobranch fishes. Canadian Journal of Fisheries and Aquatic Sciences, 74(3), pp.419-428.

Table S1 C:N ratios of tissue samples taken from both sharks and teleost prey.

|  | Fin | Muscle | Plasma | RBC |
| --- | --- | --- | --- | --- |
|  | (mean ± SD) | (mean ± SD) | (mean ± SD) | (mean ± SD) |
| *Carcharhinus amblyrhynchos* | 2.77 ± 0.11 | 3.02 ± 0.20 | 3.53 ± 0.05 | 3.16 ± 0.06 |
| *Carcharhinus albimarginatus* | 2.87 ± 0.04 | 3.01 ± 0.16 | 3.58 ± 0.03 | 3.16 ± 0.18 |
| Barracuda, Sphyraenidae |  | 3.06 ± 0.03 |  |  |
| Grouper, Serranidae |  | 3.09 ± 0.06 |  |  |
| Snapper, Lutjanidae |  | 3.09 ± 0.02 |  |  |
| Trevally, Carangidae |  | 3.09 ± 0.01 |  |  |
| Tuna, Scombridae |  | 3.09 ± 0.04 |  |  |
| Wahoo, Scombridae |  | 3.09 ± 0.02 |  |  |

RBC, red blood cells.

TABLE S2 ANOVA results for stable isotope comparisons between grey reef sharks *Carcharhinus amblyrhynchos* and silvertip sharks *Carcharhinus albimarginatus*.

| **Isotope** | **Tissue** | ***F*** | ***df*** | *P* |
| --- | --- | --- | --- | --- |
| δ^13^C | Fin | 23.890 | *1, 27* | <0.001 |
|  | Muscle | 4.488 | *1, 24* | < 0.05 |
|  | RBC | 18.660 | *1, 10* | < 0.01 |
|  | Plasma | 25.230 | *1, 10* | <0.001 |
| δ^15^N | Fin | 3.616 | *1, 27* | > 0.05 |
|  | Muscle | 6.948 | *1, 24* | < 0.05 |
|  | RBC | 9.137 | *1, 10* | < 0.05 |
|  | Plasma | 17.470 | *1, 10* | < 0.01 |
| δ^34^S | Muscle | 0.781 | *1, 24* | > 0.05 |

RBC, Red blood cells

TABLE S3 The convex hull total area (TA), standard ellipse area (SEA) and small sample size corrected SEA (SEAc) of each isotope ellipse based on the maximum likelihood estimates of the means and covariance matrices of each group.

| **Isotope** | **Tissue type** | **Metric** | ***Carcharhinus amblyrhynchos*** | ***Carcharhinus albimarginatus*** |
| --- | --- | --- | --- | --- |
| δ^13^C and δ^15^N | Fin | TA | 2.29 | 0.74 |
|  |  | SEA | 1.17 | 0.33 |
|  |  | SEAc | 1.26 | 0.36 |
|  | Muscle | TA | 7.67 | 3.17 |
|  |  | SEA | 3.06 | 1.41 |
|  |  | SEAc | 3.30 | 1.57 |
|  | RBC | TA | 1.16 | 0.09 |
|  |  | SEA | 1.31 | 0.07 |
|  |  | SEAc | 1.75 | 0.08 |
|  | Plasma | TA | 0.92 | 0.13 |
|  |  | SEA | 0.89 | 0.10 |
|  |  | SEAc | 1.18 | 0.12 |
| δ^13^C and δ^34^S | Muscle | TA | 10.43 | 12.88 |
|  |  | SEA | 3.89 | 8.22 |
|  |  | SEAc | 4.19 | 9.24 |
| δ^15^N and δ^34^S | Muscle | TA | 3.24 | 8.78 |
|  |  | SEA | 1.49 | 6.05 |
|  |  | SEAc | 1.61 | 6.81 |

Figure S1 Linear regressions examining the effect of body size of *Carcharhinus amblyrhynchos* and *Carcharhinus albimarginatus* on δ^15^N values in fin, muscle, red blood cells (RBC) and plasma. Higher δ^15^N values suggest a higher trophic level. *Carcharhinus amblyrhynchos* with pre-caudal lengths (*L*_PC_) < 80 cm were removed due to potential maternal effects. Significant relationships depicted by colour coded dashed lines and associated *R*^2^ values.

Figure S2 Linear regressions examining the effect of size of *Carcharhinus amblyrhynchos* and *Carcharhinus albimarginatus* on δ^13^C values in fin, muscle, red blood cells (RBC) and plasma. Higher δ^13^C values suggest a greater dependence on reef resources as opposed to pelagic. *Carcharhinus amblyrhynchos* with pre-caudal lengths (*L*_PC_) < 80 cm were removed due to potential maternal effects. Significant relationships depicted by colour coded dashed lines and associated *R*^2^ values.

Figure S3 Linear regressions examining the effect of size of *Carcharhinus amblyrhynchos* and *Carcharhinus albimarginatus* on δ^34^S values in muscle tissue of reef sharks. *Carcharhinus amblyrhynchos* with pre-caudal lengths (*L*_PC_) < 80 cm were removed due to potential maternal effects. No significant relationships were found.
